# The theoretical systems of OFDI location determinants in global north and global south economies

**DOI:** 10.1057/s41599-023-01597-y

**Published:** 2023-03-27

**Authors:** Yanfeng Liu, Xue Li, Xiaonan Zhu, Min-Kyu Lee, Po-Lin Lai

**Affiliations:** 1 Department of International Trade and Logistics, Graduate School, Chung-Ang University, 84 Heuksuk-Dong, Dongjak-Gu, Seoul, Republic of Korea; 2 Graduate School of Management of Technology, Pukyong National University, Busan, Republic of Korea; 3 School of Civil and Environmental Engineering, Nanyang Technological University, Singapore, Singapore; 4 Department of International Logistics, College of Business and Economic, Chung-Ang University, 84 Heuksuk-Dong, Dongjak-Gu, Seoul, Republic of Korea

**Keywords:** Economics, Economics

## Abstract

With economic transformation and industrial development, Outward Foreign Direct Investment (OFDI) from southern countries has increased rapidly. The theoretical system established by global north countries with their dominant position in the international investment market has been impacted by global south countries. The existing OFDI theory has always been based on developed countries and can only explain some international investment behavior of southern countries. The Vector Error Correction Model (VECM) is applied to conduct empirical analysis for the impact of the target country’s investment climate on the location determinants of OFDI, by applying China and the United States as example which is focusing on 172 countries from 2005 to 2019. The results reveal significant differences in the theoretical system of foreign investment between China and the United States. For China, investment climate factors such as energy, logistics infrastructure, and politics are discover as the main drivers of China’s OFDI. However, USA’s OFDI is a corporate behavior aimed at economic interests. The differences in OFDI theoretical systems and provides policy advice for northern and southern countries and departments is the major contribution of this research.

## Introduction

With the economic transformation and industrial development, Outward Foreign Direct Investment (OFDI) from emerging countries has increased rapidly (Buckley et al., 2007; Labes, 2015; Lin, 2020), Especially OFDI from emerging economies (which refers to the countries with faster economic development among southern countries) (Yakubu et al., 2020). In 2020, the OFDI of southern countries reached 392,710 million dollars, surpassing global north countries for the first time, accounting for 53.08% of the total investment in the world. As a representative of global south countries, China’s OFDI flow is 153.71 billion dollars in 2020, ranking first in the world. The stock of OFDI reached US$2.58 trillion, second only to the US (8.13 trillion dollars) and the Netherlands (3.8 trillion dollars). As a traditional powerhouse of foreign investment, the stock of the United States has always remained the first, and investment flows ranked 5th in 2020 (UNCTAD, 2021).

Since 2003, the dominant position of global north countries in international investment has been impacted by emerging economies and transition economies. In 2020, the world economic growth rate dropped to the lowest level since the international financial crisis. The growth rate of global trade in goods has slowed down significantly, and the outflow of global foreign direct investment has continued to decline (UNCTAD, 2021). COVID-19 significantly impacts OFDI in global north countries but relatively less in global south countries. Unlike most developed countries, which tend to take a more conservative attitude towards international investment, emerging and transition economies have far more aggressive outbound investment policies.

The location behavior of multinational enterprises is one of the most critical organizational considerations (Dunning, 1998; Dunning and Lundan, 2008; Buckley, 2016). Since Dunning (1958) introduced location economics to the international business domain in his first major research project, the location dimension has become an essential and distinctive element in international business research (Buckley and Ghauri, 2004). Location choice is core to the managerial decisions of multinational enterprises when engaging in OFDI. Location choice decisions in most cases are irreversible or costly to alter and hence affect the sustainable development of global enterprises (Duanmu, 2012). A location decision is very complex and involves considering multiple and diverse elements. Inconsistencies exist in the current location choice literature, and a comprehensive understanding of the factors that affect location choice is still under-developed (Kim and Aguilera, 2016; Nielsen et al., 2017). In addition, the research on the location of OFDI has always been based on developed countries. As developing economies such as China increasingly participate in OFDI, this situation is changing in recent years.

However, comparing these two groups, global south economies start their OFDI later than global north economies, and face challenging home market environments characterized by inadequate business mechanisms, political instability, and resource constraints (Casanova and Miroux, 2016). COVID-19 pandemic exacerbates the decline in investment, especially in the least northern countries and structurally weak economies (UNCTAD, 2021). As two major investment countries, the similar situation of China and the United States in the overseas investment market provides a sample for objective comparative empirical analysis of southern and northern countries. In addition, we found that China’s active promotion of overseas investment is not solely for pursuing economic results. Behind the pursuit of the economy, there is essential investment purpose such as politics, energy security, and expansion of international influence. The OFDI of the United States is market-oriented and lacks overall planning.

Although southern countries need to deal with more risks, the existing mainstream theories and frameworks of OFDI cannot reflect the actual situation of southern countries and are more applicable to developed economies. To better explain the location factors of south economies, the existing OFDI theories need to be adjusted and improved. In addition, investment determinants in southern countries are different from northern countries, and traditional OFDI theories can only explain part of the international investment behavior of emerging and transition economies. The theoretical system needs to be supplemented by actual data in southern countries. It motivates us to test our hypothesis to capture current investment data from developed and southern economies and data from investment target countries.

However, most of the existing studies focus on the support effect of specific factors, few studies focus on the research on the theoretical system of investment (Peng et al., 2008; Kang and Jiang, 2012; Chang et al., 2021), and limited studies on the theoretical system do not involve the theoretical differences between the Northern and Southern countries (Frenken and Mbuvi, 2017; Djokoto, 2021). This study aims to apply the United States and China two representative countries in global north and south economies as example to reveal the differences in OFDI theoretical systems and how FDI responds differently to host country characteristics.

The remainder of this study is described below. Section “Literature review” reviews the theoretical system of OFDI and OFDI researches. Section “Methodology” presents the research model and research data processing. The empirical analysis results are shown in section “Empirical results”. Section “Dynamic empirical analysis” analyzes the dynamic impact of location determinants on OFDI from China and the United States. Section “Conclusion” forecasts trends in investment in both the two countries. Finally, the findings, contributions, and policy implications and acknowledge the limitations of this study are discussed.

## Literature review

### Theoretical review

OFDI theory comes almost entirely from Western scholars and is based on corporate behavior in northern countries (Buckley and Casson, 1998). Hymer (1976) Explained the flow of OFDI under the imperfect markets. Lall and Siddharthan (1982) and Boddewyn (1983) examined the imperfect markets give multinational enterprises a monopoly advantage and the ability to compete with local firms in host countries. Vernon’s (1992) product life cycle theory explains why multinational corporation (MNCs) use OFDI instead of exports. Threats from competitors force companies to make foreign direct investments in product maturity.

Dunning (1998) proposed the eclectic (OLI: ownership, location, internalization) theory of international production, which is recognized as a comprehensive theory of cross-border investment. According to Dunning’s eclectic paradigm, OFDI can be divided into four types: resource seeking, efficiency-seeking, market seeking, and strategic asset seeking. This theory is widely used in the study of OFDI (Bieliński et al., 2019; Yakubu et al., 2020). However, Dunning’s eclectic paradigm cannot adequately explain current OFDI from emerging economies (Dunning, 2006; Mathews, 2006; Narula, 2006; Collinson and Rugman, 2007). Enterprises in emerging countries do not have the ownership advantages of advanced technology, high-quality brands, management knowledge as in northern countries. Therefore the ownership advantage of eclectic does not provide a vivid explanation for such investments in emerging economies. Furthermore, due to high labor costs, manufacturing costs, and high transaction costs in developed economies compared to southern economies, location and internalization advantages also do not fully explain OFDI in emerging economies (Chang et al., 2021).

As an application of the eclectic paradigm, Dunning (1981) proposed the IDP theory (investment development path), which is considered a popular theoretical approach. It is a dynamic approach to studying the relationship between economic development and OFDI. The IDP theory shows that with the development of the economy, the conditions of domestic and foreign enterprises change, which ultimately affects the inflow and outflow of OFDI. (Buckley and Casson, 1998).

Most of previous literature has applied the IDP model to study the relationship between a country’s FDI and its economic development and found that the actual development model is different from the theoretical model (Sathye, 2008; Masca and Vaidean, 2010). Narula and Guimon (2010) and Boudier-Bensebaa (2008) also pointed out that although southern countries are similar to northern countries in terms of OFDI in eastern europe countries, but are different in terms of GDP. This result also points out the difference between the empirical research and the IDP theory. The IDP theory can explain the development paths of southern countries to a minimal extent. The IDP model has always faced many limitations in empirical research (Durán and Ubeda, 2001; Satoglu, 2017), especially in southern countries (Dunning, 1986; Frenken and Mbuvi, 2017). Unlike the relationship of northern countries to IDP theory, investment development paths in southern countries deviate from IDP theory, showing evidence inconsistent with theory and experience (Andreff, 2003; Djokoto, 2021).

The resource-based theory and the basic theory of resource OFDI in emerging economies suggest that scarce, valuable, and irreplaceable resources are critical to attracting OFDI (Peteraf, 1993; Hsu and Pereira, 2008; Peng et al., 2008). On the other hand, countries with poor institutional systems lead to high transaction costs (Meyer, 2001). Governments in emerging economies provide institutional support in financial and policy incentives for companies to invest in overseas markets. Therefore, the institutional theory provides a particular explanation for the foreign direct investment of emerging economies to a certain extent (Buckley et al., 2007).

### Empirical review

In the twenty-first century, new changes have taken place in the global investment market. Global south economies that previously received OFDI began to invest heavily in international markets, The main purpose is to find overseas markets, but also to find new technologies and efficient management (Holtbrügge and Kreppel, 2012). Lecraw (1993) Unique Competitive advantages and investment incentives contribute to a particular theory of OFDI from global south countries. Compared with global north countries, MNCs from global south countries perform better in international investment (Buckley et al., 2007). As the ability to global south countries to respond to their conditions can serve as a competitive advantage in similar markets abroad, these advantages include flexibility the ability to operate with limited resources.

According to the eclectic paradigm and IDP theory, the host country’s market stimulates the inflow of OFDI, trade and foreign direct investment are exchange relationships (Horst, 2018). Economic factors such as labor, capital mobility, and human capital have significant positive implications for OFDI, both in developed and southern countries (Freckleton et al., 2012). Although the existing OFDI theories are derived from northern countries, Chang et al. (2021) tested existing theories by using stochastic frontier analysis (SFA) methods and Pooled ordinary least squares (PLOS). The results show that most existing theories apply to China’s OFDI. Janicki et al. (2005) focused on the annual bilateral FDI flows of EU-15 member countries. They found that the market size of the host country significantly increased FDI flows, while distance significantly decreased FDI flows. Foreign markets attract the OFDI of emerging economies, and market seeking is the primary driver of OFDI from emerging economies and developed countries (Duanu and Guney, 2009; Kalotay and Sulstarova, 2010; Holtbriigge and Kreppel, 2012; Das and Banik, 2015).

Access to natural resources is one of the main drivers of OFDI from developing economies (Kang and Jiang, 2012). Taking China as an example, resources seeking is an important goal of China’s OFDI (Deng, 2004). China’s OFDI in Africa is mainly focused on natural resources. In addition, the primary purpose of China’s OFDI in developed countries such as Australia is to ensure the energy demand of the domestic market (Wilson, 2011; Zhou, 2017). Obtaining high-quality natural resources from advanced economies is the main objective of OFDI from developing economies (Kalotay and Sulstarova, 2010; Beerannavar, 2013). It has become common for developing economies to acquire natural resources from developed countries. Most of them (e.g., China) strive to establish relationships with other countries in order to obtain natural resources quickly (Morck et al., 2008; Duanmu and Guney, 2009). In addition, OFDI from developing countries, especially in large economies such as China, promotes positive spillovers as the technological gap between them and firms in southern countries narrows (Battat and Aykut, 2005). They are less corporatized, less formal, and better suited to the host country’s environment than developed country models (Bhaumik and Gelb, 2005).

Logistics infrastructure is one of the critical determinants of OFDI (Khadaroo and Seetanah, 2010; Pradhan et al., 2013). Therefore, the relationship between Logistics infrastructure and OFDI is generally favorable. Countries with good logistics infrastructure will attract more OFDI inflows to improve accessibility and reduce transport costs. Improving logistics infrastructure is a crucial determinant of OFDI, especially in southern countries that lack a significant competitive advantage (Percoco, 2014; Bensassi et al., 2015; Halaszovich and Kinra, 2020). Logistics infrastructure is of outstanding importance to a country’s competitiveness as a location for investment (Arvis et al., 2010). Globalization has increased the global distribution of OFDI (Gaston and Nelson, 2002), which makes the quantity and quality of logistics infrastructure even more critical (Halaszovich and Kinra, 2020). In this context, logistics infrastructure systems are becoming increasingly important in the location determinants of OFDI (Önsel Ekici et al., 2016). It is especially true for southern countries, as transport infrastructure is a crucial determinant of attractiveness and competitiveness (Bensassi et al., 2015; Percoco, 2014). However, nearly all southern countries now face the common challenges of outdated infrastructure and underfunded (Zhang, 2015).

In addition to industry-specific factors, environmental factors such as institutional and policy environment are also important determinants of OFDI (Banik and Das, 2014); it guarantees a good business environment. Good governance and efficiency in the host country are critical for FDI, and the effectiveness of the government ensures consistency in the implementation of foreign investment policies and enhances the confidence of foreign investors (Bonnitcha, 2016; Cai et al., 2018; Mishra and Ratti, 2011). Institutional factors are also essential location determinants of OFDI (Buckley et al., 2010). Bad institutions hinder OFDI, like high taxes (Buchanan et al., 2012). As bad institutions, corrupt institutions, complex investment environment will increase the cost of investment (Mengistu and Adhikary, 2011). Interestingly, China’s OFDI is the opposite; it invests in economically and institutionally backward but resource-rich countries. Chinese multinational companies can better penetrate Asian markets because they are accustomed to operating successfully in an uncertain economic development, opaque regulatory conditions, and weak market promotion mechanisms. The effects of corruption on OFDI are blended, and most studies do not reach consistent conclusions. Some researches provide some supports for a negative link between crime and OFDI (Egger and Winner, 2006); some studies have found positive effects (Bardhan, 1997), some studies have not found any significant relationship (Campos et al., 2010).

Although the literature on OFDI in southern economies as primary recipients and OFDI from northern countries already exists, the theoretical system of OFDI and OFDI location determinants in southern economies have been rarely studied. Some studies on the OFDI location determinants from southern or northern countries have only been analyzed from a single industry perspective. The research comparing the theoretical system of location determinants of OFDI from southern and northern countries is less involved. It provides the impetus and direction for our research.

Research literature shows that almost all existing investment theories come from northern countries. However, there are significant differences in investment motives and investment characteristics between OFDI from southern and northern countries. To better explain the OFDI of MNCs in southern economies, some adjustments and refinements to the existing OFDI theories are needed. This study aims to adjust and improve the current theoretical system of FDI location determinants by comparing the theoretical systems of OFDI in the United States and China, two representative countries of developed and southern economies.

First of all, the comprehensiveness of Influencing Factors. Compared with existing research, this study uses principal component analysis to reduce a series of location determinants to analyze the theoretical systems between southern and northern countries. Provide academic reference and policy advice for OFDI from southern countries. Second, Different research methods, this study uses the VECM model to simultaneously analyze the differences in the short-term and long-term impacts of investment target countries’ environmental factors on OFDI between China and the United States. And use impulse analysis and variance decomposition to analyze the differences in the dynamics impact of the investment environment.

## Methodology

### Model specification

In summary, it becomes evident that economy, logistics, energy, and politics are critical success factors for OFDI. As they achieved the goal of the investing country, facilitated the accumulation of capital in host countries for investment, and created more employment opportunities to promote their economic development. Based on existing research such as Buckley et al. (2007); Buckley (2016); Li et al. (2018); Saidi et al. (2020); Zhao and Lee (2021). To correctly reflect the effect of independent variables on OFDI, the model is expressed as:

1 $$Y_t = EKLP$$

*Y* represents OFDI, *E* denotes economy, *K* represents energy, *L* represents logistics infrastructure, and *P* denotes politics. To remove heteroskedasticity and standardize the data, the log-linearized reduced version as follows:

2 $$lnY_t = \alpha _i + \lambda _t + \alpha lnE_{it} + \beta lnK_{it} + \gamma lnL_{it} + \partial lnP_{it} + \varepsilon _{it}$$

We empirically analyze the relationship between the investment climate of target countries and OFDI from China and the USA using the panel data regression method. *α*_*i*_ represents the cross-section fixed effect, *λ*_*t*_ denotes the time constant, and *ε*_*it*_ represents error term. We select variables from the economy, energy, logistics infrastructure, and politics to represent the investment climate of investment target countries:

*China*

3 $$\begin{array}{l}ln{\mathrm{OFDI}}_{it} = \alpha _0 + \alpha _{11}ln{\mathrm{EC}}_{it} + \alpha _{12}ln{\mathrm{EX}}_{it} + \gamma \alpha _{13}ln{\mathrm{ER}}_{it} \\\qquad\qquad\quad\, +\, \alpha \gamma _{14}ln{\mathrm{LQ}}_{it} + \alpha \gamma _{15}ln{\mathrm{LC}}_{it} + \alpha \gamma _{16}ln{\mathrm{PO}}_{it} \\\qquad\qquad\quad\, +\,\alpha _i + \lambda _t + \varepsilon _{it}\end{array}$$

*USA*

4 $$\begin{array}{l}ln{\mathrm{OFDI}}_{it} = \alpha _0 + \alpha _{21}ln{\mathrm{EC}}_{it} + \alpha _{22}ln{\mathrm{EX}}_{it} + \alpha _{23}ln{\mathrm{ER}}_{it} \\\qquad\qquad\quad\, +\,\alpha _{24}ln{\mathrm{LQ}}_{it} + \alpha _{25}ln{\mathrm{LC}}_{it} + \alpha _{26}ln{\mathrm{PO}}_{it} \\\qquad\qquad\quad\, +\,\alpha _i + \lambda _t + \varepsilon _{it}\end{array}$$

EC represents economic market, EX denotes energy export, ER represents energy resources, LQ represents logistics infrastructure quality, and LC denotes logistics capacity, PO represents politics risk. Considering serial correlation and endogeneity in the ordinary least squares estimation can lead to biased estimates (Blundell and Bond, 1998). We refer to the fully modified ordinary least square method (FMOLS) developed by Li et al. (2018) and Zhao and Lee (2021) for heterogeneous cointegrated panels. We assess the short and long-term impact of the economic, energy, logistics infrastructure, and political climate of the investment target country on China and USA’s OFDI by conducting panel-based VECM. According to the unconstrained VAR model (vector autoregressive regression) and MIC (multiple information criteria), the optimal lag order is determined, and the VECM is as follows:

5 $$\begin{array}{l}\Delta ln{\mathrm{OFDI}}_t = \partial + \alpha _{11}\Delta ln{\mathrm{EC}}_{it} + \alpha _{12}\Delta ln{\mathrm{EX}}_{it} + \alpha _{13}\Delta ln{\mathrm{ER}}_{it} \\\qquad\qquad\quad\;\;\; +\,\alpha _{14}\Delta ln{\mathrm{LQ}}_{it} + \alpha _{15}\Delta ln{\mathrm{LC}}_{it} + \alpha _{16}\Delta ln{\mathrm{PO}}_{it} \\\qquad\qquad\quad\;\;\; +\,\nu {\mathrm{ECO}}_{t - 1} + \alpha _i + \lambda _t + \varepsilon _t\end{array}$$

6 $$\begin{array}{l}{\mathrm{ECO}}_{t - 1} = ln{\mathrm{OFDI}}_{it - 1} - \partial - \alpha _{11}ln{\mathrm{EC}}_{it - 1} - \alpha _{12}ln{\mathrm{EX}}_{it - 1} \\\qquad\qquad\;\;\, -\,\alpha _{13}ln{\mathrm{ER}}_{it - 1} - \alpha _{14}ln{\mathrm{LQ}}_{it - 1} - \alpha _{15}ln{\mathrm{LC}}_{it - 1} \\\qquad\qquad\;\;\, -\,\alpha _{16}ln{\mathrm{PO}}_{it - 1}\end{array}$$

where Δ represents the first difference, ∂ denotes constant, *v* represents the revision coefficient, ECO_*t*−1_ denotes the error correction term, Formula (6) denotes the short-term relationship between OFDI and the economic, energy, logistics infrastructure, and politics variables, and Formula (5) denotes the long-term relationship.

### Data collection

OFDI varies significantly by economy, energy, logistics infrastructure, and politics factors of the investment target country. The dataset for this study consists of the panel data of the 172 investment target countries from 2005 to 2019. Panel data can provide more information and allow higher degrees of freedom than time-series and cross-sectional data (Lee and Chang, 2008). The main variables and data sources are shown in Table 1. Our dependent variable is the stock from China and the USA to the host country. The descriptive statistics of the variables are shown in Table 2.

**Table 1:** Definitions and variables source.

Variable		Definition	Source	Reference
COFDI	COFDI	Outward foreign direct investment ($ million)	Ministry of Commerce in China	Kang and Jiang (2012)
UOFDI	UOFDI	Outward foreign direct investment ($ million)	American Enterprise Institute	Camarero et al. (2021)
Economic market (EC)	PGDP	PGDP ($ million)	World Bank	Buckley et al. (2007); Ramasamy et al. (2012); Kang and Jiang (2012); Zhao and Lee (2021)
	GDP	GDP ($ million)		
	POPU	Person		
	IMPO	Imports ($)	WTO	
	EXPO	Exports ($)		
	OPEN	Openness(%)	World Bank	
Energy resources (ER)	OILR	Oil reserves (barrels)	BP Statistical Review 2020	Cheng and Ma (2007); Zhao and Lee (2021)
	GASR	Gas reserves (trillion cubic meters)		
Energy export (EX)	PFUE	Fuel exports (% of merchandise exports)	World Bank World Development Indicators	Cheng and Ma (2007); Ramasamy et al. (2012); Bevan and Estrin (2004); Zhao and Lee (2021)
	PORE	Ores and metals exports (% of merchandise exports)		
	PATE	Patent applications, residents (/)		
	PMAN	Manufacturing exports(% of merchandise exports)		
	PHTE	High-technology exports (% of manufactured exports)		
Logistics infrastructure quality (LQ)	PORQ	Quality of port infrastructure (1–7)	Global competitiveness report 2006–2020	Zhao and Lee (2021)
	ROAQ	Quality of roads (1–7)		
	AIRQ	Quality of air transport infrastructure (1–7)		
	RAIQ	Quality of railroad infrastructure (1–7)		
Logistics capacity (LC)	PTRA	Container port traffic (TEU)	World Bank World Development Indicators	Micco and Serebrisky (2006); Hong (2007); Lean et al. (2014); Bevan and Estrin (2004); Saidi et al. (2020)
	RTRA	Railways, goods transported (million ton/km)		
	ATRA	Air transport, freight (million ton/km)		
Politics risk (PO)	RULA	Rule of law (/)	Worldwide Governance Indicators	Ramasamy et al. (2012); Kang and Jiang (2012); Zhao and Lee (2021)
	COCO	Control of corruption (/)		
	GOEF	Government effectiveness (/)		
	REQU	Regulatory quality (/)		
	VOAC	Voice and accountability (/)		
	POST	Political stability and absence of violence/terrorism (/)

**Table 2:** Descriptive statistics of the variables.

	Obs	Mean	Median	Maximum	Minimum	Std. dev.
EC	2565	0.00	0.21	1.13	−4.34	1.00
EX	2565	0.00	0.32	1.53	−1.70	1.00
ER	2565	0.00	−0.48	3.31	−1.07	1.00
LQ	2565	0.00	0.17	2.13	−1.52	1.00
LC	2565	0.00	−0.19	2.35	−1.74	1.00
PO	2565	0.00	−0.14	2.21	−2.65	1.00
COFDI	2565	1.64	3.10	7.30	−3.00	3.39
UOFDI	2565	3.35	2.81	5.97	1.20	0.81

From literatures, a single variable of the economy (such as GDP, GDP per capita) is generally used to measure market potential and labor costs in empirical analysis (Liu et al., 2001; Kang and Jiang; 2012). A single variable of logistics (such as railway mileage, number of Internet users, and telephone lines) represents the regional logistics infrastructure level (Hayaloglu, 2015; Wang et al., 2020; Yang et al., 2020). However, economics, energy, logistics infrastructure, political variables should cover many aspects. A new study on OFDI now claims a host of determinants and measures that explain multinational enterprises’ location considerations (Carter et al., 1997; Oum and Park, 2004; Ekenstedt, 2004; Memedovic et al., 2008; Rodrigue, 2012).

In this research, principal component analysis methods reduce the dimensionality of large datasets on economic, energy, logistics infrastructure, and political variables. It helps improve interpretability while minimizing information loss. Another reason is that reduce multicollinearity problems, making it impossible to add all single indicators in one equation (Khan et al., 2017). Therefore, compared with a single indicator, using principal component analysis to introduce more determinants can better reflect the economy, energy, logistics infrastructure, and political situation (Sabir et al., 2019).

We first used the Kaiser-Meyer-Olkin (KMO) and Bartlett’s test to test whether the variables were suitable for principal component analysis. When the simple correlation coefficient is much larger than the partial correlation coefficient, the correlation is strong, and the KMO value is close to 1. Bartlett’s test of sphericity examines whether each investment climate variable is independent. The KMO values and Bartlett’s tests results are shown in Table 3. All KMO values are significantly greater than 0.7, and all Bartlett’s test are significantly less than 1%. The basic data on the investment climate are suitable for principal component analysis according to Kaiser (1974). In this study, the principal component analysis summarized 27 multidimensional variables into 6 one-dimensional independent variables, as shown in Table 4.

**Table 3:** KMO values and Bartlett’s tests results.

Variable		Economy	Energy	Logistics	Politics
KMO measure of sampling adequacy		0.86	0.75	0.82	0.89
Bartlett’s test of sphericity	Approx. chi-square	15211.63	11583.6	14016.04	23015.6
	df	15	28	21	15
	Sig.	0.00***	0.00***	0.00***	0.00***

*** indicate significance at 1% level.

**Table 4:** Principal component analysis results.

Total variance explained: 72.27%		EC		Total variance explained: 84.45 %		PO	
Economy	EXPO	0.95		Politics	RULA	0.98	
	IMPO	0.93			GOEF	0.96	
	PGDP	0.87			COCO	0.959	
	OPEN	0.85			REQU	0.943	
	POPU	0.82			VOAC	0.856	
	GDP	0.65			POST	0.803	
Total variance explained: 58.16%		LQ	LC	Total variance explained: 43.75%		EX	ER
Logistics	PORQ	1.01		Energy	PMAN	0.88	
	ROAQ	0.99			PORE	0.86	
	AIRQ	0.96			PFUE	0.83	
	RAIQ	0.66			PHTE	0.76	
	PTRA		0.55		PATE	0.70	
	RTRA		0.90		OILR		0.92
	ATRA		0.64		GASR		0.91

## Empirical results

The location determinants (economics, logistics, energy, politics) of different OFDI theoretical systems are listed separately. Economic, logistics infrastructures, energy resources, and policies in other geographical areas have a different impact on OFDI. Therefore, we identify the differences between China and the USA by comparing the test results between the groups.

We performed multiple tests before estimating the above models, in order to ensure the validity of the estimates and avoid the possibility of spurious regressions. Since the panel unit root tests have higher power than the unit root tests (Breuer et al., 2002; Gutierrez, 2006; Mahadevan and Asafu-Adjaye, 2007). The first step is to test the stationarity of the data by using the panel unit roots test. Then to avoid spurious regressions, we perform a co-integration test for the long-run equilibrium relationship between variables (Yuan and Kuang, 2010). Panel cointegration allows for heterogeneity and reduces variables’ collinearity compared to traditional co-integration analysis (Mahadevan and Asafu-Adjaye, 2007). Our ultimate aim is to use the VECM to measure the relationship between the investment climate and OFDI in the short and long term.

### Panel unit root test

The correlation matrix is shown in Table 5. We first perform panel unit root tests on the variables to detect whether they are stable and further avoid spurious regression problems (Li et al., 2018). The panel unit root tests involve two categories, the Breitung t-statistic, LLC’s test, and the IPS statistic are for the same root process tests; PP-Fisher and ADF-Fisher Chi-square test are for different root process tests (Dickey and Fuller, 1979; Breitung, 2001; Levin et al., 2002; Im et al., 2003). We employed four analyses and presented the results in Table 6. The results confirm that all the variables of China and the USA are significant at the 1% level.

**Table 5:** Correlation matrix results.

	COFDI	UOFDI	JING5	NENG5	NENG6	WU5	WU6	ZHENG
COFDI	1.00							
UOFDI	−0.12	1.00						
EC	0.12	0.00	1.00					
EX	0.02	0.14	0.51	1.00				
ER	0.19	0.08	0.17	0.09	1.00			
LQ	0.00	0.18	0.38	0.58	0.05	1.00		
LC	−0.11	0.19	0.44	0.57	0.28	0.42	1.00	
PO	−0.29	0.16	0.25	0.44	−0.23	0.53	0.34	1.00

**Table 6:** Panel unit root test results.

	LLC	IPS	ADF-Fisher	PP-Fisher
cofdi	−3.30***	−17.69***	332.92***	1184.43***
uofdi	−3.81***	−20.35***	409.30***	1282.51***
EC	−6.68***	−16.00***	321.77***	1366.39***
EX	−11.60***	−20.92***	471.33***	1380.27***
ER	−12.13***	−15.59***	308.13***	1236.77***
LQ	−16.86***	−21.94***	455.57***	1431.68***
LC	−10.65***	−14.11***	245.42***	1353.15***
PO	−16.37***	−24.62***	534.95***	1491.04***

The probability of LLC and IPS tests assume asymptotic normality, Only Fisher’s test is computed using the asymptotic chi-square distribution.

*** indicates statistical significance at the 1% level.

### Panel co-integration analysis

We conducted a panel co-integration analysis to test the long-term equilibrium relationship between China and the USA’s OFDI and the investment environment variables of the target country. We performed a co-integration test using the Kao (1999) test, panel co-integration test results are shown in Table 7. The co-integration test results of China and the USA significantly reject the null hypothesis that there is no co-integration relationship. It shows that there is a long-term equilibrium relationship between the China and the USA’s OFDI and the investment climate of the investment target countries.

**Table 7:** Panel co-integration test results.

Country		*t*-statistic	Prob.
China	ADF	3.59***	0.00
	Residual variance	11.54	
	HAC variance	0.23	
USA	ADF	−5.50***	0.00
	Residual variance	1.26	
	HAC variance	0.03	

*** indicates statistical significance at the 1% level.

### Empirical analysis results

#### Empirical analysis results of China

The empirical analysis results between China’s OFDI and the investment climate of the target countries are shown in Table 8. The lagged EC (error correction)’s negatively significant coefficients confirm that the adjustment rate of OFDI from shock toward the long-term stability is 0.947 in China’s model. The coefficients for most all variables are significantly in line as expected. The results for energy and logistics infrastructure are interesting and additionally provide some novel insights. It differs from the empirical results in the USA. Empirical results of China’s OFDI show that energy, logistics infrastructure, and politics are essential components of China’s OFDI system (Chang, 2014; Hajzler, 2014; Kohl, 2019), but different from Buckley et al. (2007) and Cheung and Qian (2009), the economy is not reflected.

**Table 8:** VECM estimates analysis results (China).

Variable	Coefficient	Std. error	*t*-statistic
EC	0.126	0.234	0.541
EX	0.235**	0.082	2.876
ER	1.760***	0.225	7.806
LQ	−0.279**	0.112	−2.493
LC	−0.215**	0.101	−2.138
PO	−0.268	0.328	−0.816
D(EC)	−0.012	0.170	−0.069
D(EX)	0.211***	0.063	3.319
D(ER)	1.789***	0.156	11.476
D(LQ)	−0.273***	0.088	−3.110
D(LC)	−0.235***	0.085	−2.751
D(PO)	−0.578**	0.225	−2.572
Lagged EC(−1)	−0.947***	0.059	−16.047

*** indicate significance at 1% level.

Energy export (EX) and energy resources (ER) positively impact China’s OFDI in the short and long term. It proves that China’s OFDI is biased towards countries with energy export and energy resources in both the short and long term (Cheung and Qian, 2009; Hayakawa et al., 2013; Chang, 2014; Hajzler, 2014). The logistics capacity (LC) and logistics quality (LQ) negatively impact China’s OFDI in the short- and long term. It proves that China’s OFDI is biased towards countries with logistics capacity and logistics quality in both the short- and long-term (Iwanow and Kirkpatrick, 2009, Solmecke, 2016, Kohl, 2019). The politics (PO) has a substantial and negative impact on OFDI in the short time at the 5% significance level, proving that China’s OFDI prefers countries with high political risks in the short term. Compared with economic markets, China focuses more on energy and infrastructure investment. One possible explanation is that logistics infrastructure is a prerequisite for energy development. China can build new logistics infrastructure in countries with weak logistics capabilities, while it can repair infrastructure in countries with poor logistics infrastructure quality.

#### Empirical analysis results of USA

The empirical analysis results between USA’s OFDI and the investment climate of the target countries are shown in Table 9. The lagged EC(error correction)’s negatively significant coefficients confirm that the adjustment rate of OFDI from shock toward the long-term stability is 0.982 in USA’s model. In the coefficient of the variables, the results are different from China’s OFDI. The short-term contribution of the economy (EC) to the USA’s OFDI is positive. Nonetheless, energy resources have a significant adverse effect on OFDI both in the long and short term at a substantial level of 5%. The results show that the USA’s OFDI aimed at the economic market is different from China. Resource intensity is not a significant factor. This finding contradicts our expectations. It may be a consequence of the sample composition since resource-rich countries, such as African countries, have a minimal presence in the sample of the USA’s OFDI. Alternatively, it may be that the resource seeking is an industry-specific attraction for the USA’s OFDI, but not so strongly as to affect aggregate USA’s OFDI, which contains a broad range of industries. Unlike the USA’s marketization model (commodity dumping), China’s OFDI model is based on the investment climate such as energy and logistics infrastructure and politics that can actively attract China’s OFDI. Empirical results show that economics is an integral part of the US investment system (Chenaf-Nicet and Rougier, 2016; Holtbriigge and Kreppel, 2012; Das and Banik, 2015). energy, logistics, and politics have no significant impact. USA’ OFDI is only interested in economic factors, unlike China’s OFDI model, which focuses on energy, logistics, and politics at the same time.

**Table 9:** VECM estimates analysis results (USA).

Variable	Coefficient	Std. error	*t*-statistic
EC	0.02	0.02	0.94
EX	0.00	0.01	−0.32
ER	−0.04**	0.02	−2.15
LQ	0.01	0.01	1.09
LC	−0.02	0.02	−0.74
PO	−0.02	0.04	−0.55
D(EC)	0.04**	0.01	2.55
D(EX)	0.00	0.01	−0.30
D(ER)	−0.04**	0.02	−2.42
D(LQ)	0.02	0.01	1.58
D(LC)	0.00	0.01	0.26
D(PO)	−0.03	0.04	−0.81
Lagged EC (−1)	−0.98***	0.04	−25.27

**, and *** indicate significance at 5%, and 1%, respectively.

## Dynamic empirical analysis

### Panel Granger causality test

**Fig. 1:**
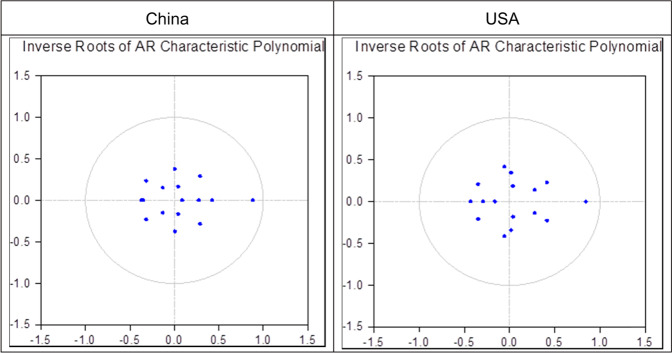
Inverse roots of AR characteristic polynomial for China and USA.

To further analyze the causal relationship between the investment climate of target countries and China and the USA’s OFDI, panel Granger causality and inverse roots of AR characteristic polynomial were used to test the variables of China and the USA. As Fig. 1 shows, they are all in the unit circle, and all inverse roots of China and the USA are less than 1, proving that the empirical model is stable.

Table 10 shows a causality relationship between China’s OFDI and the variable group excluding EX. In the USA’s OFDI, the results are the same as those of China, and there has a granger causality relationship between OFDI and the variable group excluding LC.

**Table 10:** VAR Granger causality test results.

	Excluded	Chi-sq
China	EC	25.315***
	EX	1.331
	ER	17.140***
	LQ	75.826***
	LC	14.802***
	PO	12.537***
	All	154.751***
USA	EC	82.522***
	EX	11.045***
	ER	7.140***
	LQ	53.744***
	LC	3.598
	PO	7.351**
	All	204.435***

**, and *** indicate significance at 5%, and 1%, respectively.

### Impulse response analysis

While the VECM model is good at accounting for short and long-term effects between variables, it cannot account for dynamic effects over time. We performed impulse response analysis and variance decomposition analysis to overcome this problem. The impulse response function measures the impact of the model variables in response to shocks in one or more variables. The purpose of impulse analysis is to analyze dynamic effects, which are the limitations of the VECM model. Examining the dynamic impact of the economy, energy, logistics infrastructure, and political environment on Chinese and U.S. foreign direct investment elucidates the current and future impact of standard deviation shocks in the investment climate on OFDI. The shock of the investment environment is illustrated for ten periods, and the impulse response function curve is obtained.

**Fig. 2:**
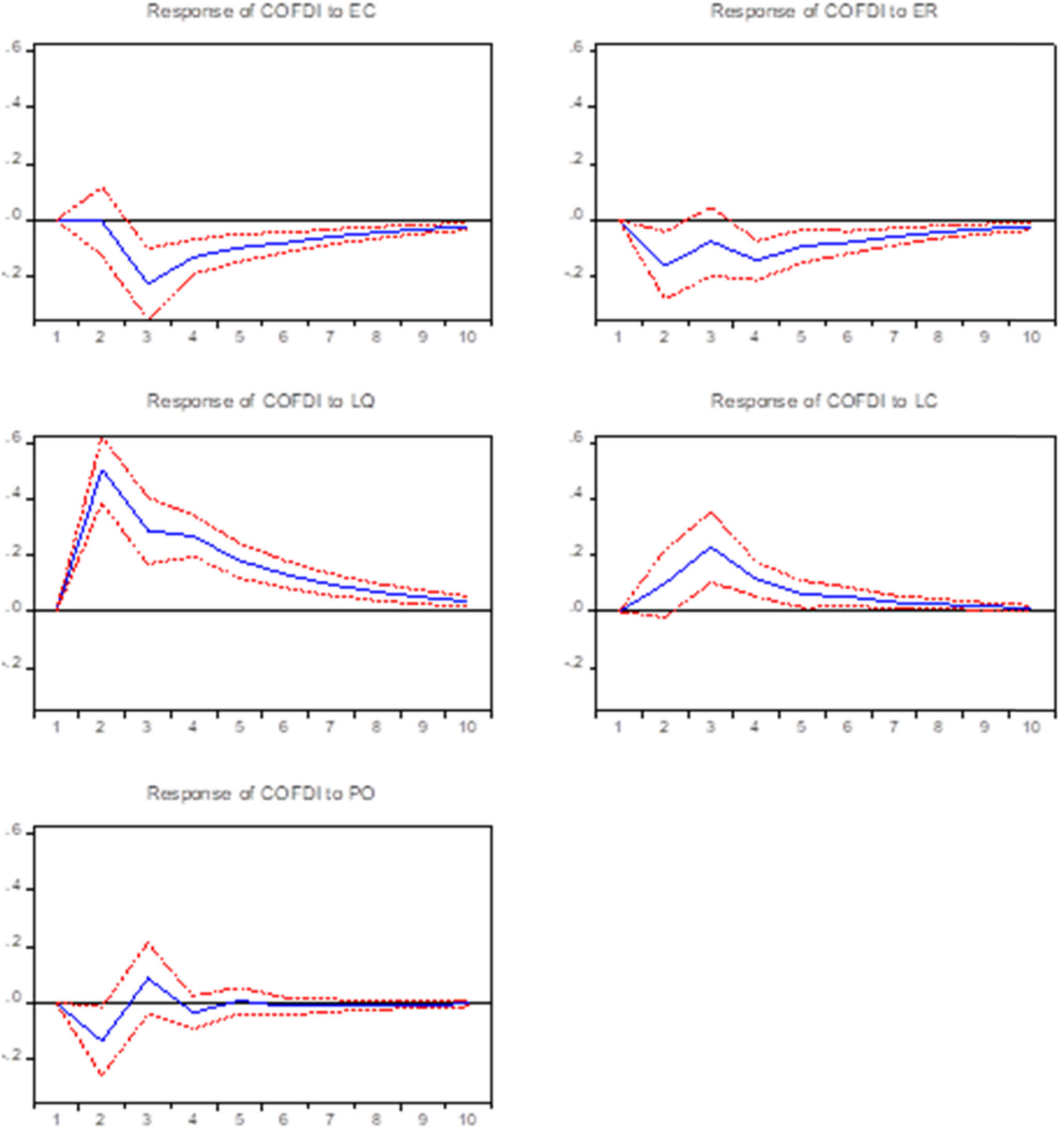
Impulse response functions results for China.

As shown in Fig. 2, China’ OFDI has a significant lag. A one standard deviation shock from the EC and ER can negatively affects OFDI in the long term. A one standard deviation chock from the LQ and LC shows a positive effect on OFDI in the long term. PO has a weak negative impact. It shows that the economics variable (EC) and energy variables (ER) hurt OFDI, LQ, and LC positively affect OFDI in the long term. Overall, PO hurts OFDI are consistent with the empirical analysis results.

**Fig. 3:**
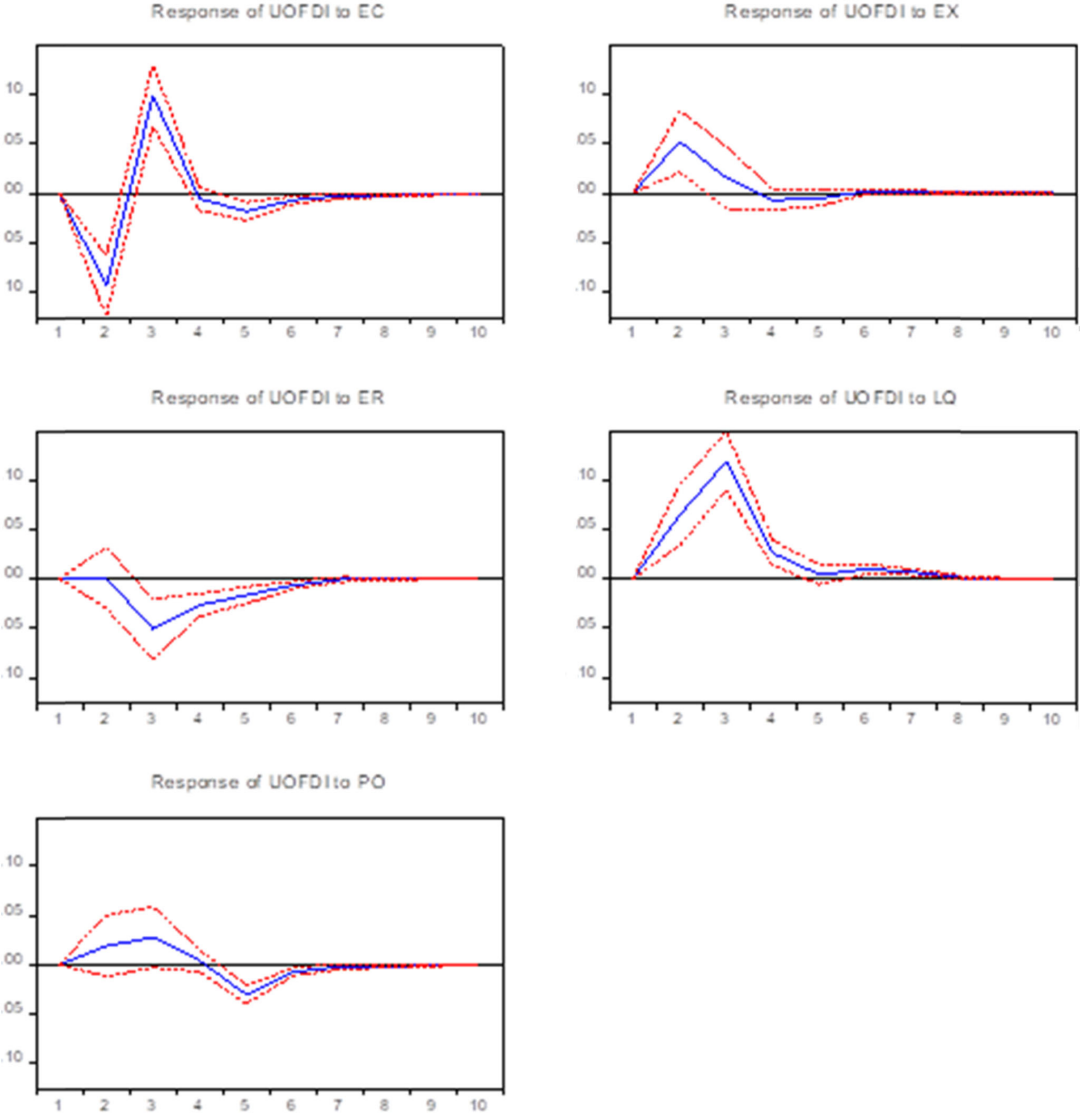
Impulse response functions results for USA.

USA’s OFDI has a significant lag as shown in Fig. 3, OFDI has a negative impact in the long term when subjected to a one standard deviation shock from ER. In addition, OFDI shows a positive effect in the long term with A one standard deviation shock from EX and LQ. With a one standard deviation shock from ER, OFDI reacted to the minimum value immediately and then increased rapidly. The third phase reached the maximum positive impact. Then it turned into a weak negative impact again, then began to decline gradually. The PO has a weak influence on OFDI. It is positive in the early period and negative in the middle period and reaches the maximum negative value in the 5th period, after which the effect gradually decreases. Overall, ER shows a negative impact, LQ shows a positive effect on OFDI consistent with the empirical analysis results.

### Variance decomposition analysis

Variance decomposition is an analytical method for measuring the relative importance of individual variables in the model. The variance decomposition provides information about the relative importance of random innovation. This decomposes the variance of each variable’s prediction error into components that can contribute to each endogenous variable. This is useful for assessing how shocks reverberate through the system to assess external shocks to each variable (Brahmasrene et al., 2014). Variance decomposition analyzed the strength of the relationship between investment variables by examining the variance contribution rate of each structural shock to OFDI, in contrast to impulse response analysis. We set the period to 10, the variance decomposition results are shown in Tables 11 and 12.

**Table 11:** Variance decomposition results (China).

	COFDI	EC	EX		ER		LQ			
Period	COFDI	EC	EX	EC	ER	COFDI	LQ	EC	EX	
1	100.00	98.16	71.91	28.07	91.24	6.12	62.16	16.93	20.00	
5	93.06	95.09	70.27	27.71	86.63	6.34	60.37	16.65	18.25	
10	92.54	95.09	70.23	27.71	86.53	6.39	60.15	16.63	18.16	
	**LC**				**PO**					
**Period**	**LC**	**EC**	**EX**	**ER**	**PO**	**COFDI**	**EC**	**EX**	**ER**	**LQ**
1	55.14	22.10	14.99	5.49	54.78	9.54	8.65	10.97	4.51	10.45
5	53.76	21.81	15.15	5.74	53.60	9.58	8.49	10.78	5.85	10.25
10	53.76	21.81	15.15	5.74	53.60	9.58	8.49	10.78	5.85	10.26

**Table 12:** Variance decomposition results (USA).

	UOFDI	EC	EX		ER	LQ			
Period	UOFDI	EC	EX	EC	ER	LQ	UOFDI	EC	EX
1	100.00	99.99	72.15	25.96	96.15	63.56	2.32	14.11	20.00
5	92.59	94.24	70.04	25.57	89.45	60.04	5.12	13.50	17.76
10	92.54	94.23	70.02	25.57	89.45	59.97	5.14	13.52	17.74
	**LC**				**PO**				
**Period**	**LC**	**UOFDI**	**EC**	**EX**	**PO**	**EC**	**EX**	**ER**	**LQ**
1	59.24	3.16	18.95	14.68	57.74	5.24	11.64	9.07	11.22
5	57.07	5.05	18.46	14.37	55.51	5.16	11.29	10.32	11.02
10	57.06	5.05	18.46	14.37	55.50	5.16	11.29	10.31	11.02

In China’s OFDI, OFDI and EC’s volatility is primarily affected by itself. However, the variance contribution rate of the EC to EX declines from 28.07 to 27.71%. EC also has a solid ability to explain EX. The variance contribution rate of EC and EX to LQ is more than 15%, showing that EC and EX have a more substantial explanatory power for LQ. the variance contribution rate of ER, EX, and EC to LC are more than 5%, 15%, and 20%, respectively, indicating that ER has insufficient explanatory power, EX has a more substantial explanatory power, and EC has a strong explanatory power to explain LC. EX and LQ’s variance decomposition are more than 10%, indicating that EX and LQ have a more substantial explanatory power for PO. All other investment variables’ contribution rate is less than 10%, meaning explanatory power is weak.

In the USA, the variance decomposition of UOFDI, EC, and ER drop to 92.54, 94.23, and 89.45%, shows that their volatility is primarily affected by themselves. The variance decomposition rate of EC to EX is 25.57%, which indicating that EC has a strong ability to explain EX. The variance decomposition rate of UOFDI, EC, and EX to LQ is more than 5%, 10%, respectively, indicating that UOFDI has a weak explanatory power. EC and EX have a more substantial explanatory power to explain LC. The variance decomposition rate of UOFDI, EC, and EX to LC is more than 5% and 10%, respectively, indicating that UOFDI has a weak explanatory power. EC and EX have a more substantial explanatory power to explain LC. The rate of variance decomposition of EC, EX, ER, and LQ to PO is more than 5% and 10%, respectively, indicating that EC has a weak explanatory power. EX, ER, and LQ have a more vital descriptive ability to explain PO. The variance contribution rates of other investment climate variables are all less than 5%, indicating that their explanatory power is insufficient.

## Conclusion

OFDI from global south countries is increasingly prominent in the international investment market, especially in emerging countries. However, the theoretical system of OFDI originated in northern countries and always has been studied based on the investment behavior of global north countries. There are significant differences in the investment motives and investment characteristics of OFDI from global north and south countries. To better explain OFDI from global south countries, it is necessary to make some adjustments and refinements to the existing OFDI theories on the location determinants.

In this study, the short- and long-term impacts of the target country’s investment environment (economy, logistics, energy, and politics) on location determinants of OFDI from China and the USA are examined. The results show that there is a big difference in the theoretical system of foreign investment between China and the United States (Buckley et al., 2010; Kim and Aguilera, 2016; Nguyen, 2021). USA’s OFDI is a corporate behavior aimed at economic benefits (Casanova and Miroux, 2016). The difference is that China’s OFDI is a long-term development plan led by the Chinese government to improve logistics infrastructure and develop energy and benefit from these development projects. According to research conclusions, the global north countries can learn from the investment experience of the United States, with the market as the determining factor. In contrast, China’s investment experience with the purpose of infrastructure construction and energy development provides a reference for most global south countries. For example, investment target countries could prioritize investment in different areas, industries, and sectors. The transportation sector of global south countries can prioritize attracting China’s OFDI to build their lagging infrastructure, the energy sector can introduce capitalˏ equipment and technical support for energy exploitation and export (Percoco, 2014; Bensassi et al., 2015; Zhang, 2015). The research results prove that economic factors such as the domestic and international markets are the most important determinants of US investment, and countries with weak logistics infrastructure quality and capabilities are more attractive to Chinese investment. Therefore, the global north countries can introduce Chinese capital to repair or build new infrastructure while maintaining economic activities with the United States. In addition, the research results show that target countries with high levels of energy reserves and energy exports are the most attractive for Chinese investment. Therefore, cooperation in the energy field effectively diversifies energy dependence, whether for China or the investment destination country.

Some limitations of this study need to be acknowledged. First, this study was conducted with China and the United States as examples. Since the policies and implementation of foreign investment vary from country to country and region, it is impossible to generalize the theory to other countries directly. Therefore, follow-up research should be conducted in different countries or cultural contexts to cross-validate the conclusions of the analysis, possibly yielding interesting findings. Second, we did not examine the spillover effects of OFDI and did not include a spatial correlation analysis of the model. External shocks (such as COVID-19) are also not fully considered. Hence, future research aims to establish models at different geographical levels to test the regional spillover effects under external shocks.

## Data Availability

The datasets generated during and/or analyzed during the current study are available from the corresponding author on reasonable request.
